# 

**Published:** 1878-06

**Authors:** 


### CONTENTS:

ORIGINAL LECTURES:
On Dr. Brown-Sequard and his recent Lectures. By J. S. Jewell, M. D....... 561
ORIGINAL COMMUNICATIONS:
Precocious and other Phenomena of Sexual Orgasm. By J. N. Hyde, M. D. 527
Cases of Purpura Hæmorrhagica, with Observations on the Pathology and Treatment of the Disease. By N. S. Davis, M. D. ........ 585
Cases of Lithotrity; an Instrument for Finding small Remaining Fragments of Stone by Auscultation. By E. Andrews, M. D. ........ 592
A Case of Ovariotomy. By Wm. Meacher, M. D. ........ 598
Arsenic in Subnitrate of Bismuth. By J. H. Salisbury, M. D. ........ 601
CLINICAL REPORTS:
Notes from Private Practice:
Fibroid Tumor of Uterus successfully Treated by Hypodermic Injections of Ergotine........ 604
Superfœtation, or Twin Conception? 605
SOCIETY REPORTS:
Twenty-eighth Annual Meeting of the Illinois State Medical Society........ 607
CORRESPONDENCE:
Vital Statistics of the Sandwich Islands. 613
Bidding for Pauper Practice........ 618
How to Measure the Relative Length of the Lower Limbs........ 621
REVIEWS AND BOOK NOTICES........ 624
Books and Pamphlets Received........ 630
SUMMARY:
Syphilis........ 631
Dermatology........ 637
Surgery........ 638
Therapeutics........ 641
Practical Medicine........ 642
TRANSLATION:
On Certain Sensitive Troubles of Mesocephalic Origin........ 646
MEDICAL NEWS AND ITEMS........ 649
ANNOUNCEMENTS FOR THE MONTH........ 672

